# New Books

**Published:** 2005-08

**Are Chemical Journals Too Expensive and Inaccessible?: A Workshop Summary to the Chemical Sciences Roundtable**

Ned D. Heindel, Tina M. Masciangioli, Eva von Schaper, eds.

Washington, DC:National Academies Press, 2005. 50 pp. ISBN: 0-309-09590-5, $18

**Data Analysis and Visualization in Genomics and Proteomics**

Francisco Azuaje, Joaquin Dopazo, eds.

Hoboken, NJ:John Wiley & Sons, Inc., 2005. 284 pp. ISBN: 0-470-09439-7, $150

**Does the Built Environment Influence Physical Activity? Examining the Evidence—Special Report 282**

Committee on Physical Activity, Health, Transportation, and Land Use

Washington, DC:National Academies Press, 2005. 268 pp. electronic report available via PDF, free

**Encyclopedic Reference of Genomics and Proteomics in Molecular Medicine**

Detlev Ganten, Klaus Ruckpaul, eds.

New York:Springer-Verlag, 2005. 1,500 pp. ISBN: 3-540-44244-8, $499

**Energy and Environment**

Richard Loulou, Jean-Philippe Waaub, Georges Zaccour, eds.

New York:Springer-Verlag, 2005. 282 pp. ISBN: 0-387-25351-3, $79.95

**Estimating the Contributions of Lifestyle-Related Factors to Preventable Death: A Workshop Summary**

Planning Committee on Estimating the Contributions of Lifestyle-Related Factors to Preventable Death

Washington, DC:National Academies Press, 2005. 80 pp. ISBN: 0-309-09690-1, $18

**From Resource Scarcity to Ecological Security: Exploring New Limits to Growth**

Dennis Pirages, Ken Cousins, eds.

Cambridge, MA:MIT Press, 2005. 280 pp. ISBN: 0-262-16231-8, $60

**Guide to Analysis of DNA Microarray Data, 2nd ed.**

Steen Knudsen

Hoboken, NJ:John Wiley & Sons, Inc., 2005. 160 pp. ISBN: 0-471-65604-6, $39.95

**Health Effects of Transport-Related Air Pollution**

Michal Krzyzanowski, Birgit Kuna-Dibbert, and Jürgen Schneider, eds.

Geneva:World Health Organization, 2005. 206 pp. ISBN: 92-890-1373-7, $54

**Implications of Genomics for Public Health: Workshop Summary**

Lyla Hernandez, ed.

Washington, DC:The National Academies Press, 2005. 98 pp. ISBN: 0309096073, $18

**Introduction to Bioethics**

John Bryant

Hoboken, NJ:John Wiley & Sons, Inc., 2005. 256 pp. ISBN: 0-470-02197-7, $125

**Metabolome Analyses: Strategies for Systems Biology**

Seetharaman Vaidyanathan, George G. Harrigan, Royston Goodacre, eds.

New York:Springer-Verlag, 2005. 383 pp. ISBN: 0-387-25239-8, $129

**Modeling Biological Systems: Principles and Applications, 2nd ed.**

James W. Haefner

New York:Springer-Verlag, 2005. 480 pp. ISBN: 0-387-25011-5, $79.95

**Proteomics of Spermatogenesis**

G.S.Gupta

New York:Springer-Verlag, 2005. 837 pp. ISBN: 0-387-25398-X, $179

**RNA Silencing**

Esra Galun

Hackensack, NJ:World Scientific Publishing Co., 2005. 468 pp. ISBN: 981-256-206-0, $78

**Stem Cells: From Bench to Bedside**

Ariff Bongso, Eng Hin Lee, eds.

Hackensack, NJ:World Scientific Publishing Co., 2005. 700 pp. ISBN: 981-256-126-9, $112

**The Carcinogenic Effects of Polycyclic Aromatic Hydrocarbons**

Andreas Luch, ed.

Hackensack, NJ:World Scientific Publishing Co., 2005. 740 pp. ISBN: 1-86094-417-5, $178

**Viral Genome Packaging: Genetics, Structure, and Mechanism**

C.E. Catalano, ed.

New York:Springer-Verlag, 2005. 164 pp. ISBN: 0-306-48227-4, $139

**World Health Statistics 2005**

World Health Organization

Geneva:World Health Organization, 2005. 95 pp. ISBN: 92-4-159326-1, $36

